# Layered immune biomarker monitoring in IgA nephropathy: from mucosal IgA dysregulation to precision therapeutic response assessment

**DOI:** 10.3389/fmed.2026.1917560

**Published:** 2026-09-04

**Authors:** Yuanyue Lu, Changxi Sun, Xiaoshuang Zhou

**Affiliations:** 1 Department of Nephrology, Tianjin Fifth Central Hospital (Peking University Binhai Hospital), Tianjin, China; 2 Department of Nephrology, Shanxi Provincial People’s Hospital, The Fifth Clinical Medical College of Shanxi Medical University, Taiyuan, China; 3 Shanxi Kidney Disease Institute, Taiyuan, China

**Keywords:** APRIL, BAFF, biomarker, Gd-IgA1, immune complex, targeted therapy

## Abstract

IgA nephropathy (IgAN) is the most common primary glomerular disease worldwide and remains a major cause of chronic kidney disease and kidney failure. Conventional monitoring still depends largely on proteinuria, estimated glomerular filtration rate (eGFR), hematuria, blood pressure and biopsy-based pathological classification. These measures are clinically indispensable, but they mainly capture downstream kidney injury and may lag behind the mucosal and systemic immune events that drive active disease. In the targeted-therapy era, this limitation has become more consequential. Targeted-release budesonide, BAFF/APRIL dual inhibitors, APRIL inhibitors and complement-directed therapies act at different levels of the IgAN immunopathogenic cascade. Galactose-deficient IgA1 (Gd-IgA1) remains central to disease biology, yet its standalone value as a marker of disease activity or prognosis is limited by assay heterogeneity, ethnic and clinical variability, treatment exposure and differences in histopathological context. Increasing evidence suggests that the pathogenicity of IgA depends not only on Gd-IgA1 concentration, but also on mucosal origin, polymeric state, autoantibody binding, immune-complex formation, mesangial retention and complement activation. This Review synthesizes recent advances in immune biomarker monitoring in IgAN, with emphasis on mucosal immune activity, Gd-IgA1, polymeric IgA, IgA-containing immune complexes, complement activation and kidney injury biomarkers. Its contribution is not another undifferentiated catalog of candidate markers. Instead, biomarkers are organized according to their position in the disease cascade and their relationship to treatment mechanism: upstream mucosal immune activation, pathogenic IgA immune-complex burden, complement-mediated intrarenal amplification and tissue injury, and conventional clinical outcomes. The framework is intended to support mechanistic research, trial enrichment and longitudinal pharmacodynamic assessment, while distinguishing established clinical measures from biomarkers that remain investigational. Its use for treatment selection or routine precision monitoring requires prospective validation and assay standardization.

## 1 Introduction

IgA nephropathy is defined by mesangial IgA-dominant immune-complex deposition, but its clinical course is highly variable, ranging from isolated microscopic hematuria to persistent proteinuria, declining kidney function and kidney failure (1). Even with optimized supportive care, many patients remain at measurable risk of progression. The KDIGO 2025 guideline places greater emphasis on early risk recognition, optimized supportive therapy and stricter proteinuria control, with a target below 0.5 g/day and ideally below 0.3 g/day (2). This change reflects a more proactive approach to IgAN management.

The multi-hit model remains the dominant framework for IgAN pathogenesis. It starts with excess production of circulating Gd-IgA1, followed by anti-glycan autoantibody formation, circulating IgA-containing immune complexes, mesangial deposition, complement activation and renal injury (1, 3, 4). This model has been highly productive, particularly for therapeutic development. Still, circulating Gd-IgA1 alone does not account for the heterogeneity seen in clinical practice. Some patients with high Gd-IgA1 progress slowly, and reported correlations with proteinuria, hematuria, histological activity and long-term kidney outcome are inconsistent (5–8). Experimental work also suggests that increased circulating Gd-IgA1 is not necessarily sufficient to produce glomerular IgA deposition unless the appropriate mucosal and immunological context is present (9).

At the same time, IgAN treatment is moving beyond supportive care. Targeted-release budesonide is designed to act on gut-associated lymphoid tissue and reduce pathogenic IgA production (10, 11). Telitacicept and atacicept inhibit B-cell activating factor (BAFF) and a proliferation-inducing ligand (APRIL), targeting B-cell and plasma-cell pathways involved in Gd-IgA1 and autoantibody production (12–15). APRIL-specific agents such as zigakibart focus more selectively on IgA generation and plasma-cell survival (16), whereas complement inhibitors act further downstream after immune-complex deposition (17). These drugs intervene at different points in the cascade, but routine follow-up still relies mainly on proteinuria and eGFR.

Accordingly, the central monitoring question is not limited to who will progress. It also concerns whether modifiable immune activity persists, whether a targeted therapy engages its intended pathway and whether early biological changes precede later clinical benefit. The contribution of this Review is therefore not another inventory of candidate biomarkers. We organize them by biological position—mucosal immune activation, pathogenic IgA immune-complex burden, intrarenal complement and tissue injury, and clinical outcomes—and align each layer with the mechanism and timing of targeted treatment. This framework is intended to guide mechanistic studies, trial enrichment and longitudinal pharmacodynamic assessment. Its use for baseline risk stratification, selection of targeted therapy or prediction of long-term eGFR slope remains investigational and requires prospective validation.

## 2 Current clinical monitoring and its limitations

Proteinuria, estimated glomerular filtration rate (eGFR), hematuria, blood pressure and kidney histology remain the practical foundation of IgAN assessment. Persistent proteinuria is among the strongest predictors of long-term outcome and remains a major treatment target and an established surrogate endpoint in clinical trials. Serial eGFR and eGFR slope provide complementary information on chronic progression, whereas hematuria may indicate ongoing glomerular inflammatory activity in some patients. These conventional measures remain indispensable for routine treatment decisions and clinical trials (2, 18). Current supportive care, including renin-angiotensin system (RAS) blockade and sodium-glucose cotransporter 2 (SGLT2) inhibition where appropriate, remains directed at reducing proteinuria and slowing kidney-function loss.

Their principal limitation is not a lack of clinical relevance, but limited biological specificity. Proteinuria may result from active immune-complex deposition, glomerular inflammation and altered filtration-barrier permeability, but it may also persist because of segmental sclerosis, glomerulosclerosis or tubulointerstitial fibrosis. It therefore reflects both potentially modifiable inflammatory injury and established structural damage rather than serving as a direct histological measure of fibrosis. Similarly, eGFR decline generally represents accumulated nephron loss and may become apparent only after upstream immunological injury has been present for some time. Neither measure alone can reliably distinguish active immune-mediated disease from predominantly chronic, less reversible injury.

Kidney biopsy remains the diagnostic reference standard for IgAN and provides essential prognostic and mechanistic information through the Oxford MEST-C classification and tissue immunostaining. Its main limitations are invasiveness, sampling variability, procedure-related risk and dependence on the timing of tissue acquisition. Repeated biopsy is not appropriate for routine longitudinal monitoring. Nevertheless, repeat biopsy may be clinically considered in selected patients with suspected disease reactivation, an atypical change in clinical course or a prolonged interval from the initial biopsy when renewed immunomodulatory treatment is being considered.

The gap between pathway activity and conventional clinical readouts has become more relevant with the introduction of targeted therapies. Targeted-release budesonide, telitacicept, atacicept and zigakibart intervene primarily in mucosal immune activation, B-cell or plasma-cell signaling and pathogenic IgA production, whereas proteinuria and eGFR mainly capture downstream consequences. A more informative monitoring strategy should therefore retain conventional clinical endpoints while adding mechanism-matched biomarkers that assess mucosal immune activation, pathogenic IgA immune-complex burden, complement activation and kidney injury.

## 3 Mucosal origins of nephritogenic IgA: rationale for upstream monitoring

Mucosal immune dysregulation is one of the most characteristic features of IgAN. Episodes of gross hematuria after upper respiratory or gastrointestinal infection point to a close link between mucosal antigenic stimulation and disease activity (1, 19). Most IgA is generated in mucosa-associated lymphoid tissues, including gut-associated, nasal-associated and tonsillar lymphoid tissue. When these responses become dysregulated, Gd-IgA1-producing B cells or plasma cells may enter the circulation and contribute to pathogenic immune-complex formation.

Recent multi-omics work has made this concept more concrete. A 2026 Kidney International study suggested that terminal ileum-derived IgA+β7+ cells may be an important cellular source of pathogenic IgA in human IgAN. Ileocecal IgA in patients was enriched for polymeric and Gd-IgA1 O-glycoforms, and terminal ileal IgA+ B cells showed abnormalities in O-glycosylation-related enzymes. Circulating IgA+ B cells in IgAN resembled terminal ileum-derived IgA+ B cells more closely than those from the ascending colon or tonsils (20). These findings give biological support to the idea that gut-derived pathogenic IgA signatures could be monitored, at least in research settings.

From a practical monitoring perspective, IgA+β7+ B cells and related mucosal-homing phenotypes could in principle be assessed in peripheral blood by multiparameter flow cytometry or mass cytometry. However, this approach is not yet a routine clinical assay for IgAN because antibody panels, gating strategies, pre-analytical handling and clinically meaningful cutoffs remain to be standardized. At present, it should be viewed as a mechanistic and trial-enrichment tool rather than a ready-to-use biomarker for routine outpatient follow-up.

The gut is unlikely to be the only relevant mucosal compartment. In murine IgAN, nasal-associated lymphoid tissue has been identified as a dominant induction site for aberrantly glycosylated IgA after TLR9 stimulation. Cells derived from this compartment produced more nephritogenic IgA and IgG-IgA immune complexes than cells from mesenteric lymph nodes or Peyer’s patches, and nasal CpG-oligodeoxynucleotide stimulation worsened kidney injury (21). Mucosal origin may therefore be compartment-specific and may vary by species, antigenic trigger and immune context.

Innate immune signaling provides another link between mucosal stimulation and Gd-IgA1 production. Gut dysbiosis, impaired intestinal barrier function and enrichment of Escherichia-Shigella have been associated with activation of TLR4/MyD88/NF-κB signaling, B-cell stimulatory factors and Gd-IgA1 production (22). TLR9 activation can promote abnormal IgA glycosylation through APRIL- and IL-6-dependent pathways and increase IgG-IgA immune-complex formation (23). These observations place active IgAN upstream of the kidney and support monitoring strategies that include mucosal immune activation, B-cell stimulation and pathogenic IgA generation.

## 4 Gd-IgA1 as a biomarker: important but insufficient alone

Galactose-deficient IgA1 is the most extensively studied IgAN biomarker. Systematic reviews and meta-analyses consistently report higher levels in IgAN than in healthy controls and many disease controls (5, 6), supporting its diagnostic and mechanistic relevance. A prognostic signal has also been reported. In one prospective cohort of 275 patients, higher serum Gd-IgA1 was independently associated with a greater risk of kidney function deterioration (7).

The prognostic role of Gd-IgA1 nevertheless remains unsettled. A 2024 systematic review found no consistent association between serum Gd-IgA1 and validated prognostic factors, although an inverse relationship with kidney function was observed. Other studies have reported weak or inconsistent associations with proteinuria, hematuria and Oxford MEST-C lesions (8, 24). Assay platform, cohort composition, sampling time, treatment exposure, ethnicity and disease stage are probable contributors to these discrepancies. Additional biological heterogeneity may also be relevant, including differences in the mucosal source and polymeric state of IgA, anti-glycan autoantibody binding, immune-complex formation and clearance, mesangial retention, intrarenal complement activation and the relative contributions of active inflammation and chronic structural damage. The wide variation in clinical course is therefore unlikely to be explained by circulating Gd-IgA1 concentration alone.

Histopathological context may also modify the prognostic meaning of Gd-IgA1. In patients whose kidney biopsy is dominated by active lesions, such as mesangial or endocapillary hypercellularity and cellular crescents, a high Gd-IgA1 level may more plausibly reflect ongoing modifiable immune activity. By contrast, when segmental sclerosis and tubular atrophy/interstitial fibrosis predominate, proteinuria and eGFR decline may be driven mainly by irreversible chronic damage, so the same circulating Gd-IgA1 value may show a weaker relationship with subsequent outcome. Future studies should therefore test Gd-IgA1 dynamics together with Oxford MEST-C activity and chronicity patterns rather than evaluating it as an isolated cross-sectional marker.

Mechanistic data also caution against overinterpreting Gd-IgA1 concentration alone. In a human IgA1 mouse model, B cell-specific C1galt1 knockout markedly increased circulating Gd-IgA1 but did not lead to substantial glomerular IgA deposition under physiological or inflammatory conditions. Conversely, mucosa-derived IgA1 from patients with IgAN induced stronger mesangial deposition than serum- or myeloma-derived IgA1 despite similar or lower Gd-IgA1 content (9). Thus, Gd-IgA1 appears necessary but not sufficient for pathogenicity; tissue origin, polymeric state, autoantibody binding and immune-complex formation are likely to be decisive.

Measurement remains another limitation. Most clinical studies have relied on lectin-based ELISA, which provides useful information but cannot fully describe the complexity of IgA glycosylation. High-resolution LC-MS has shown that both O- and N-glycosylation features of serum IgA1 and IgA2 are associated with IgAN and glomerular function, and glycopeptide-based signatures may outperform lectin-based Gd-IgA1 assays (25). Gd-IgA1 should therefore be viewed as an entry point into immune monitoring rather than a standalone measure of disease activity.

## 5 Polymeric IgA and IgA-containing immune complexes: candidate markers of pathogenic immune-complex burden

The pathogenic relevance of IgA in IgAN depends not only on circulating Gd-IgA1 concentration but also on molecular form and the capacity to form immune complexes. Gd-IgA1 may be recognized by IgG or IgA anti-glycan antibodies, generating circulating complexes with a tendency toward mesangial retention. Polymeric IgA may have greater pathogenic potential than monomeric IgA because of its larger size, altered clearance, multivalent receptor interactions and greater capacity to form macromolecular complexes (1, 3, 26).

Human evidence comes from clinically distinct settings. In a transplantation cohort, pre-transplant levels of Gd-IgA1, IgA-IgG complexes and IgA-soluble CD89 complexes were associated with recurrent IgAN after kidney transplantation, with reported areas under the receiver operating characteristic curve of 0.86, 0.82, and 0.78, respectively (27). These findings support a relationship between immune-complex characteristics and recurrence, but they should not be extrapolated directly to disease activity in native-kidney IgAN.

Trial-embedded and treatment-response analyses provide preliminary longitudinal evidence. Targeted-release budesonide was associated with reductions in Gd-IgA1, total IgA and polymeric IgA, and an early decrease in polymeric IgA was associated with a later reduction in proteinuria (11, 28). Telitacicept studies likewise reported reductions in Gd-IgA1 and IgA-containing immune complexes, with changes in immune-complex measures showing associations with proteinuria response (12, 29). These observations suggest that polymeric IgA and IgA-containing immune complexes may provide pharmacodynamic information beyond Gd-IgA1 alone.

The evidence remains exploratory. Available studies include transplantation cohorts, small observational series and secondary biomarker analyses within therapeutic studies. Assay platforms are not standardized, clinically actionable thresholds have not been established and no prospective study has shown that treatment decisions guided by these markers improve outcomes. Polymeric IgA and IgA-containing immune complexes should therefore be regarded as candidate indicators of pathogenic immune-complex burden and treatment-associated biological change rather than validated measures of disease activity or predictors of clinical response.

## 6 Complement activation and kidney injury biomarkers

Once IgA-containing immune complexes are deposited in the mesangium, complement activation can amplify local inflammation and tissue injury. The alternative and lectin pathways appear particularly relevant in IgAN, whereas classical pathway activation is less prominent. Glomerular deposition of mannose-binding lectin (MBL), MBL-associated serine proteases (MASPs), C4d and C3 has been associated with more severe proteinuria, hematuria and histological injury (17, 30).

For monitoring, complement biomarkers occupy a position between immune-complex formation and overt clinical injury. Gd-IgA1 and polymeric IgA describe upstream or intermediate pathogenic load; complement markers ask a different question–whether deposited immune complexes have triggered inflammatory amplification within the kidney. This distinction is becoming clinically relevant as complement inhibitors move into IgAN trials and practice. Patients with strong complement activation may form a subgroup more likely to benefit from complement-directed treatment.

A further distinction is needed between systemic and intrarenal complement readouts. Serum C3 and C4 concentrations and the serum IgA/C3 ratio are inexpensive and widely available, but they may be influenced by systemic synthesis and consumption and therefore may not accurately reflect glomerular complement activation. Urinary complement fragments may be more proximal to intrarenal inflammatory activity and can be repeatedly sampled, but their interpretation is affected by urine concentration, proteinuria and assay-platform variability. Tissue deposition of C3, C4d, MBL, and MASP provides the most direct anatomical evidence of local activation, although it remains limited by the invasiveness, sampling variability and timing of biopsy. Serum, urinary and tissue complement markers should therefore be viewed as complementary rather than interchangeable measurements (17, 31).

Clinical development of lectin-pathway inhibition also illustrates the need for caution. Narsoplimab, a monoclonal antibody targeting MASP-2, was safe and well-tolerated in a small phase 2 study and was associated with a potential reduction in proteinuria (32). However, the randomized, double-blind, placebo-controlled phase 3 ARTEMIS-IgAN trial (NCT03608033) was terminated after the prespecified interim analysis did not meet the primary efficacy endpoint (33). This result underscores that biological pathway relevance does not necessarily translate into clinical efficacy and that complement biomarkers are not yet validated for selecting patients or predicting response to lectin-pathway inhibition.

Urinary kidney injury biomarkers add another layer. Kidney injury molecule-1 (KIM-1), neutrophil gelatinase-associated lipocalin (NGAL), monocyte chemoattractant protein-1 (MCP-1) and epidermal growth factor (EGF) are not specific to IgAN, but they capture tubular injury, inflammation and repair responses that may connect immune activity with later kidney function decline. Urinary interleukin-18 (IL-18) has also emerged as a potential prognostic biomarker. In a prospective multicenter cohort of 136 patients with IgAN, urinary IL-18 levels above 28.1 pg/mg creatinine were associated with a 4.7-fold higher adjusted risk of progression, defined as a greater than 50% decline in eGFR or end-stage kidney disease, after adjustment for clinical variables and Oxford MEST-C scores (34). Because this threshold was cohort-derived and the study included 28 outcome events, external validation, assay harmonization and evaluation of incremental clinical value remain necessary before routine use.

## 7 Monitoring response to targeted therapies

### 7.1 Targeted-release budesonide

Targeted-release budesonide provides clinical proof of principle that modifying mucosal immune activity can improve IgAN outcomes. In the phase 3 NefIgArd trial, treatment reduced the urinary protein-to-creatinine ratio (UPCR) and was associated with preservation of eGFR (10). Biomarker analyses from NEFIGAN showed changes in Gd-IgA1, polymeric IgA, and IgA-containing immune complexes (11). A 2025 Clinical Kidney Journal study further reported that early reduction in polymeric IgA during the first 2 months was associated with proteinuria reduction at 6 months (28). These findings make Gd-IgA1 and polymeric IgA plausible response markers for mucosal-targeted therapy, although their decision thresholds remain undefined.

### 7.2 BAFF/APRIL dual inhibition

B-cell activating factor and APRIL support B-cell activation, plasma-cell survival, IgA class switching, Gd-IgA1 production and autoantibody generation (35, 36). Telitacicept and atacicept target this pathway and have shown effects on pathogenic IgA biomarkers as well as proteinuria.

Telitacicept reduced circulating Gd-IgA1 and IgA-containing immune complexes in IgAN, and changes in immune-complex measures were associated with proteinuria reduction (12). A 2026 interim analysis of a phase 3 trial reported that 39 weeks of telitacicept led to a greater reduction in 24-h UPCR than placebo in high-risk IgAN; high risk in this context referred to biopsy-proven IgAN with persistent proteinuria despite stable, optimized RAS blockade, such as 24-h UPCR ≥ 0.8 g/g or total proteinuria ≥ 1.0 g/day (13). Atacicept phase 2b and long-term extension studies showed sustained reductions in Gd-IgA1, hematuria and UPCR, with stable eGFR slopes (14, 15). A phase 3 interim analysis also reported a greater reduction in UPCR with atacicept than placebo at week 36 (37).

For BAFF/APRIL inhibitors, monitoring should not focus only on proteinuria. Total IgA, Gd-IgA1, polymeric IgA, IgA immune complexes, IgG, IgM and safety-related immunoglobulin changes are all relevant. The key unanswered question is whether early reduction in IgA immune-complex burden can reliably predict later proteinuria reduction and eGFR stabilization.

### 7.3 APRIL inhibition

A proliferation-inducing ligand-specific inhibition offers a more focused approach to suppressing IgA production and plasma-cell survival. Zigakibart, an anti-APRIL monoclonal antibody, reduced IgA, Gd-IgA1, and IgM levels and was associated with proteinuria reduction and eGFR stabilization in a phase 1/2 study (16). This mechanism provides a coherent setting in which to evaluate free APRIL, total IgA, Gd-IgA1, polymeric IgA, IgA-containing immune complexes and broader immunoglobulin profiles as pharmacodynamic markers. Their value for treatment selection or prediction of clinical response has not been established.

### 7.4 Complement inhibitors

Complement inhibitors act downstream of IgA production, at the stage of inflammatory amplification after immune-complex deposition. Their response assessment should therefore rely less on Gd-IgA1 or polymeric IgA and more on complement activation markers, urinary complement fragments, glomerular C3/C4d/MBL deposition and downstream kidney injury markers (17). Complement-based stratification may eventually help identify patients most likely to benefit from this class of therapy.

A therapy-oriented summary of candidate response markers is provided in Table 2. In practice, the biomarker panel should follow the drug mechanism: upstream IgA-axis markers are most informative for mucosal or BAFF/APRIL-directed therapy, whereas complement and tubular-injury markers are more relevant for complement inhibition.

## 8 A layered immune monitoring framework for IgAN

Current evidence can be organized into a four-layer monitoring framework.

The first layer is upstream mucosal immune activity. Candidate readouts include Gd-IgA1, APRIL, BAFF, IL-6, IgA+β7+ B cells, mucosal homing receptors and gut microbiome-related markers. This layer is intended to capture ongoing immune activation and pathogenic IgA generation.

The second layer is pathogenic IgA immune-complex burden. Polymeric IgA, IgA-IgG immune complexes, IgA-sCD89 complexes and other IgA-containing immune complexes belong here. These markers may provide additional information on immune-complex formation and early pharmacodynamic change beyond Gd-IgA1 alone, but they remain investigational.

The third layer is renal inflammatory amplification and tissue injury. C3, C4d, MBL, MASP, urinary complement fragments, KIM-1, NGAL, MCP-1, EGF, and IL-18 reflect the transition from immune-complex deposition to local inflammation, tubular injury and parenchymal damage.

The fourth layer remains the clinical outcome layer: proteinuria, hematuria, eGFR, eGFR slope, 40% eGFR decline and kidney failure. These endpoints remain indispensable for clinical decisions, trials and regulatory evaluation.

The practical value of this framework lies in interpretation rather than immediate prescription. A patient with an early fall in polymeric IgA or IgA immune complexes but delayed proteinuria improvement may still be showing a biologically meaningful upstream response. Conversely, persistent immune-complex elevation despite stable proteinuria may suggest residual immunological activity and future risk. Nevertheless, this layered framework should be viewed as a testable conceptual model rather than a validated clinical algorithm. Its utility will require prospective evaluation against proteinuria trajectories, eGFR slope, Oxford MEST-C lesions and established risk-prediction tools.

### 8.1 Potential clinical use cases and current boundaries

The framework separates several potential use cases that should not be treated as equivalent. Baseline immune profiling may characterize whether IgA-axis activation, immune-complex formation or complement-mediated injury is detectable, but it is not yet validated for routine risk classification or for choosing one targeted therapy over another. Proteinuria, eGFR, hematuria, blood pressure, kidney histology and established risk-prediction tools remain the basis of clinical decision-making.

The most immediate application is trial-embedded mechanistic and pharmacodynamic assessment. For mucosal or BAFF/APRIL-directed therapy, serial changes in Gd-IgA1, polymeric IgA or IgA-containing immune complexes can be tested as indicators of target engagement before maximal proteinuria reduction occurs. For complement-directed therapy, urinary or tissue complement measures and kidney injury markers are more closely aligned with treatment mechanism. These changes should be evaluated against later proteinuria trajectories, hematuria and eGFR slope rather than interpreted as independent clinical endpoints.

**FIGURE 1:**
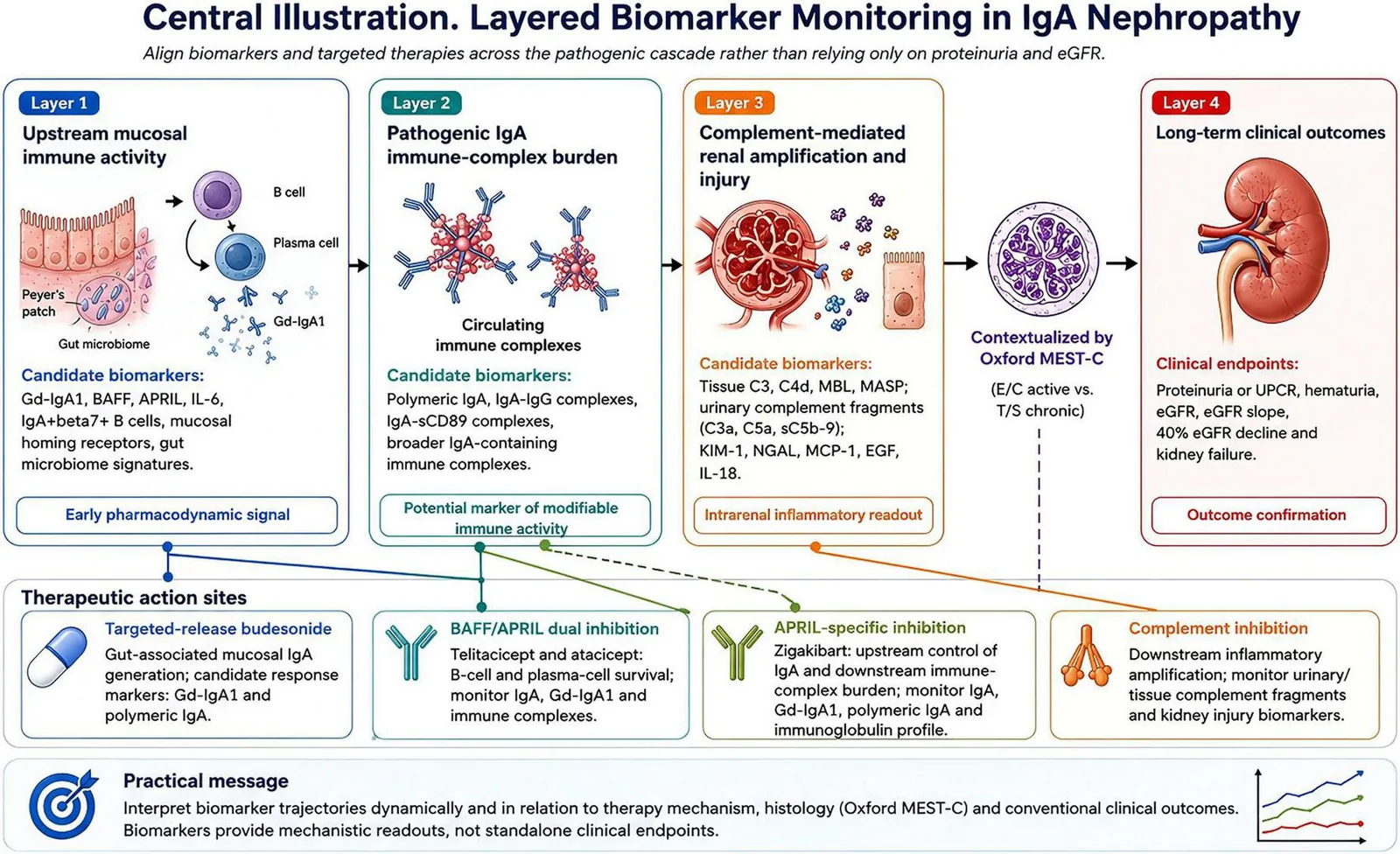
Layered biomarker framework for monitoring IgA nephropathy in the targeted-therapy era. The framework aligns candidate biomarkers and therapeutic mechanisms across four levels: upstream mucosal immune activity, pathogenic IgA immune-complex burden, complement-mediated intrarenal amplification and kidney injury, and long-term clinical outcomes. Targeted-release budesonide mainly acts on mucosal IgA generation; telitacicept and atacicept act on BAFF/APRIL-mediated B-cell and plasma-cell pathways; zigakibart more selectively suppresses APRIL-driven IgA production; and complement inhibitors act downstream at the level of complement amplification. Biomarkers should be interpreted dynamically and in relation to treatment mechanism, histology and conventional clinical outcomes. The figure is conceptual: evidence level and current clinical readiness are summarized in Table 1, and therapy-specific monitoring candidates are summarized in Table 2. APRIL, a proliferation-inducing ligand; BAFF, B-cell activating factor; C3a, complement 3a; C4d, complement 4d; C5a, complement 5a; EGF, epidermal growth factor; eGFR, estimated glomerular filtration rate; Gd-IgA1, galactose-deficient IgA1; IL-6, interleukin-6; IL-18, interleukin-18; KIM-1, kidney injury molecule-1; MASP, mannose-binding lectin-associated serine protease; MBL, mannose-binding lectin; MCP-1, monocyte chemoattractant protein-1; NGAL, neutrophil gelatinase-associated lipocalin; sC5b-9, soluble terminal complement complex; sCD89, soluble CD89; UPCR, urinary protein-to-creatinine ratio.

Long-term prognostic use requires a separate standard of evidence. Candidate biomarkers should demonstrate incremental value beyond the International IgAN Prediction Tool and Oxford MEST-C pathology through external validation, calibration and assessment of clinical utility. Figure 1 shows the biological organization of the four layers; Table 1 distinguishes their clinical readiness, and Table 2 summarizes therapy-matched monitoring candidates and the present level of evidence.

**TABLE 1:** Evidence level and clinical readiness of candidate biomarkers for IgAN monitoring.

Biomarker or assessment domain	Specimen/platform	Evidence level / current clinical use	Main limitation or interpretive boundary
Proteinuria, hematuria, eGFR and blood pressure	Routine urine and blood tests; clinical measurement	Established clinical standard; essential for treatment decisions, prognostic assessment and trial endpoints	Downstream readouts; cannot reliably distinguish active immune-mediated injury from chronic structural damage
Oxford MEST-C classification	Kidney biopsy; light microscopy	Established diagnostic and prognostic pathology framework	Invasive and dependent on sampling and biopsy timing; unsuitable for frequent longitudinal reassessment
International IgAN Prediction Tool	Clinical and pathological variables at or after kidney biopsy	Validated prognostic tool for baseline risk estimation and trial stratification	Predicts long-term progression rather than current immune-pathway activity or pharmacodynamic response
Tissue IgA, C3, C4d, MBL, and MASP deposition	Kidney biopsy; immunofluorescence or immunohistochemistry	Selected pathological assessment supported mainly by observational biopsy cohorts	Requires biopsy; staining methods and prognostic thresholds are not fully standardized
Serum Gd-IgA1	Serum; lectin-based ELISA, monoclonal antibody-based assays or glycoproteomics	Clinical/translational biomarker; investigational for longitudinal monitoring	Assay heterogeneity, ethnic and disease-stage variation, inconsistent prognostic associations and no validated treatment threshold
Polymeric IgA and IgA-containing immune complexes	Serum or plasma; specialized research assays	Exploratory clinical-trial or pharmacodynamic biomarker	Small cohorts, platform heterogeneity, limited external validation and no actionable cutoff
IgA-IgG and IgA-soluble CD89 complexes	Serum or plasma; specialized immune-complex assays	Exploratory recurrence, risk-enrichment or mechanistic biomarker	Limited availability, cost, assay reproducibility and uncertain transferability between recurrent and native-kidney IgAN
BAFF, APRIL and IL-6	Serum or plasma immunoassays	Investigational mechanistic or pharmacodynamic biomarker	Circulating levels may not reflect tissue activity; target-binding therapies may complicate assay interpretation
IgA+β7+ B cells and other mucosal-homing phenotypes	Peripheral blood; multiparameter flow cytometry or mass cytometry	Mechanistic and early translational biomarker; possible trial-enrichment use	Antibody panels, gating strategies, specimen handling and clinically meaningful thresholds are not standardized
Serum complement measures, including C3, C4, and IgA/C3 ratio	Routine serum assays	Readily available but non-specific supportive measures	May not reflect intrarenal activation and are influenced by systemic synthesis and consumption
Urinary complement fragments	Urine assays for C3a, C5a, soluble C5b-9 and related fragments	Investigational intrarenal pathway biomarker	Affected by urine concentration, proteinuria, non-specific filtration and assay platform
Urinary KIM-1, NGAL, MCP-1, EGF, and IL-18	Urine immunoassays or multiplex platforms	Investigational kidney injury or prognostic biomarker	Not specific to IgAN; cohort-derived thresholds and incremental clinical value require external validation

Evidence categories are descriptive and do not represent a formal GRADE assessment. “Established clinical standard” indicates routine use supported by clinical practice and guideline-based evidence; “clinical/translational” and “investigational” indicate human evidence that remains insufficient for routine treatment decisions. APRIL, a proliferation-inducing ligand; BAFF, B-cell activating factor; eGFR, estimated glomerular filtration rate; EGF, epidermal growth factor; Gd-IgA1, galactose-deficient IgA1; IgAN, IgA nephropathy; IL-6, interleukin-6; IL-18, interleukin-18; KIM-1, kidney injury molecule-1; MASP, MBL-associated serine protease; MBL, mannose-binding lectin; MCP-1, monocyte chemoattractant protein-1; NGAL, neutrophil gelatinase-associated lipocalin.

**TABLE 2:** Therapy-matched candidate biomarkers and current evidence for treatment-response monitoring in IgA nephropathy.

Therapeutic strategy	Target level and candidate response markers	Evidence/current use status	Interpretation and limitation
Targeted-release budesonide	Gut-associated mucosal IgA production; total IgA, Gd-IgA1, polymeric IgA, IgA-containing immune complexes and UPCR	Trial-embedded exploratory pharmacodynamic evidence; UPCR and eGFR remain established clinical endpoints	Early IgA-axis changes may indicate target engagement, but decision thresholds and prospective response rules are not validated
BAFF/APRIL dual inhibition Telitacicept; atacicept	B-cell activation and plasma-cell survival; IgA, Gd-IgA1, polymeric IgA, IgA immune complexes, IgG, IgM, and UPCR	Clinical-trial pharmacodynamic evidence; immunoglobulin monitoring also informs safety	Biomarker changes have been associated with proteinuria response but are not validated for treatment selection or response prediction
APRIL-specific inhibition Zigakibart and related agents	IgA class switching and plasma-cell pathways; free APRIL, total IgA, Gd-IgA1, polymeric IgA, immune complexes and immunoglobulin profile	Early clinical-trial pharmacodynamic evidence	Mechanistically aligned markers require standardized assays and prospective validation against clinical outcomes
Complement-directed therapy	Complement amplification after immune-complex deposition; serum, urinary or tissue complement markers, KIM-1, NGAL and UPCR	Complement biomarkers remain investigational; clinical endpoints remain the standard	Gd-IgA1 may remain unchanged; pathway markers should be matched to the drug target and interpreted with kidney injury and clinical outcomes
Supportive and kidney-protective therapy	Hemodynamic and chronic-injury pathways; RAS blockade, SGLT2 inhibitors and endothelin-pathway agents; proteinuria, eGFR slope and blood pressure	Routine clinical care with established monitoring measures	These measures are essential for risk and response assessment but do not directly quantify upstream immune activity

APRIL, a proliferation-inducing ligand; BAFF, B-cell activating factor; eGFR, estimated glomerular filtration rate; Gd-IgA1, galactose-deficient IgA1; IgAN, IgA nephropathy; KIM-1, kidney injury molecule-1; NGAL, neutrophil gelatinase-associated lipocalin; RAS, renin-angiotensin system; SGLT2, sodium-glucose cotransporter 2; UPCR, urine protein-to-creatinine ratio.

## 9 Discussion: controversies, clinical translation and future directions

### 9.1 Remaining controversies in immune biomarker monitoring

Several controversies need to be acknowledged before immune biomarker monitoring can be translated into routine IgAN care. First, serum Gd-IgA1 is mechanistically central but clinically incomplete: it distinguishes IgAN from many controls in aggregate, yet its correlations with proteinuria, hematuria, histological activity and long-term outcome vary across cohorts (5–8). Second, circulating immune markers may not fully reflect intrarenal events. A patient may have measurable systemic IgA-axis activation without active glomerular inflammation, whereas established glomerulosclerosis or tubulointerstitial fibrosis can sustain proteinuria despite partial immune suppression. Third, complement biomarkers are compartment-dependent. Serum C3, C4, or IgA/C3 ratios are accessible but non-specific, while urinary complement fragments and tissue C3, C4d, MBL, or MASP deposition may better capture local complement activation but are less standardized and less widely available (17, 31).

These controversies argue against replacing proteinuria, eGFR and biopsy-based risk assessment with any single immune biomarker. A more defensible approach is to interpret immune markers as pathway-level information. In this view, Gd-IgA1 reflects a permissive upstream substrate, polymeric IgA and IgA-containing immune complexes reflect a more proximal pathogenic load, and complement or tubular injury markers reflect whether immune-complex deposition has translated into renal inflammatory injury.

### 9.2 Assay standardization and clinical feasibility

Clinical feasibility differs widely across biomarkers. Proteinuria, eGFR, blood pressure, hematuria and standard biopsy immunofluorescence are already embedded in clinical practice. Serum immunoglobulin profiles and serum complement levels are also widely accessible, although they are not IgAN-specific. Some clinical laboratories now offer Gd-IgA1-related testing, including serum biomarker screening and tissue KM55 immunostaining (38, 39). By contrast, Gd-IgA1 assays, polymeric IgA quantification, IgA-containing immune-complex assays, urinary complement fragments and high-resolution glycoproteomic signatures remain limited by platform differences, reagent variability, reference standards and uncertain thresholds. Candidate biomarkers should therefore be separated by practical readiness rather than presented as equally mature clinical tools.

For regulatory and clinical use, future studies will need harmonized assays, inter-laboratory reproducibility data, agreed reporting units, biologically plausible thresholds and longitudinal response definitions. Biomarker panels should also be evaluated by discrimination, calibration, reclassification and decision-curve analyses to determine whether they add value beyond proteinuria, eGFR, MEST-C and the International IgAN Prediction Tool.

An additional methodological consideration is the definition of healthy control groups. Incidental mesangial IgA deposition has been reported in asymptomatic donor kidneys, including Japanese and Chinese cohorts, although whether all such cases represent latent IgAN remains uncertain (40, 41). Diagnostic biomarker and genetic case-control studies should therefore apply careful clinical phenotyping and acknowledge the possibility of control misclassification, particularly in populations with a relatively high background prevalence of subclinical IgA deposition.

### 9.3 Integration with pathology and clinical risk prediction

Immune biomarker interpretation is likely to depend substantially on histopathological context. Active lesions, particularly endocapillary hypercellularity (E1) and cellular or fibrocellular crescents (C1/C2), may indicate ongoing inflammatory injury in which elevated immune-pathway biomarkers are more likely to reflect potentially modifiable disease activity. In contrast, tubular atrophy/interstitial fibrosis (T1/T2), together with segmental sclerosis, indicates established structural damage and may weaken the relationship between circulating immune activity and current proteinuria or subsequent recovery. The clinical meaning of any individual lesion should therefore be interpreted together with kidney function, proteinuria and treatment exposure.

Biopsy timing is also important. Oxford MEST-C findings represent the pathological state at the time of tissue acquisition rather than a permanent disease phenotype. When several months of supportive care separate biopsy from the initiation of immune-directed therapy, active lesions may resolve, persist or evolve, while chronic injury may accumulate. Consequently, a biopsy performed years earlier may not reliably establish the presence or absence of current modifiable inflammation. Repeat biopsy is not suitable for routine monitoring, but may be clinically justified in selected patients with suspected reactivation, unexpected clinical deterioration or a prolonged interval from the original biopsy when renewed immunomodulatory treatment is being considered.

Biomarker-guided monitoring should therefore be evaluated as an addition to, not a substitute for, pathology and established clinical risk tools. A practical future model may combine baseline Oxford MEST-C features and conventional risk prediction with dynamic immune markers measured before and after targeted therapy. Such an approach could help distinguish early biological response, delayed clinical response, persistent pathway activity and progression driven predominantly by irreversible chronic damage.

The International IgAN Prediction Tool provides validated baseline risk estimation from clinical and histological variables available at or after kidney biopsy (42). It addresses the probability of long-term progression rather than whether an immune pathway is currently active or whether a treatment has engaged its target. Dynamic biomarkers should therefore be evaluated for incremental value beyond this tool, with prespecified assessment of discrimination, calibration, risk reclassification and clinical utility, and with attention to whether performance differs between active and chronic Oxford MEST-C patterns.

### 9.4 Future development of biomarker-guided precision therapy

The most useful next step is not simply to discover more biomarkers, but to validate dynamic biomarker patterns in clinically meaningful settings. Trial-embedded biomarker studies can define whether early reductions in polymeric IgA, IgA immune complexes or complement activation predict later UPCR response and eGFR preservation. Real-world cohorts can test whether such patterns remain informative across heterogeneous patients, different background supportive therapies and variable biopsy chronicity. Target trial emulation may also help evaluate whether biomarker-guided treatment escalation or switching improves outcomes compared with conventional proteinuria-based monitoring alone.

Future panels will probably need to be mechanism-specific. Mucosal-targeted and BAFF/APRIL-directed therapies should be evaluated mainly with IgA-axis and immune-complex markers, whereas complement inhibitors should be monitored using complement and renal injury readouts. This mechanism-matched strategy is more biologically coherent than using the same biomarker set for every therapy.

### 9.5 Limitations of the current evidence and this review

This article is a narrative synthesis rather than a systematic review, and study selection was not based on a prespecified search strategy or formal evidence-grading process. The biomarker literature is heterogeneous with respect to ethnicity, disease stage, biopsy timing, background supportive therapy, immunosuppressive exposure, specimen handling and outcome definitions. Direct comparison across studies and biomarkers is therefore limited.

Evidence for polymeric IgA, IgA-containing immune complexes, mucosal-homing B-cell populations, urinary complement fragments and kidney injury markers is derived largely from small observational cohorts, transplantation studies or exploratory analyses embedded in clinical trials. Several targeted-therapy datasets are recent, interim or secondary biomarker analyses, and many assays lack reference standards, inter-laboratory reproducibility and clinically actionable thresholds. Associations with treatment response do not establish that biomarker-guided treatment decisions improve outcomes. The proposed framework should therefore be considered a structure for hypothesis generation and prospective validation, not a clinical practice algorithm.

### 9.6 Conclusion

IgAN treatment is entering an era of targeted immune intervention, but clinical monitoring still relies heavily on downstream markers such as proteinuria and eGFR. Gd-IgA1 remains central to disease biology and biomarker research, yet it is not sufficient as a standalone marker of disease activity or treatment response. Polymeric IgA, IgA-containing immune complexes, complement activation markers and kidney injury biomarkers may provide more pathway-specific information, particularly when interpreted alongside pathology and conventional clinical risk assessment.

A layered immune monitoring model offers a useful conceptual bridge between IgAN pathogenesis and precision therapy. Its role, however, should remain appropriately cautious: it is a framework for hypothesis generation, trial design and longitudinal validation, not a replacement for established clinical endpoints. In the targeted-therapy era, the central question is not only who will progress, but which patients have modifiable immune activity and whether treatment is suppressing the relevant pathogenic pathway early enough to change long-term outcome.

## References

[1] Stamellou E Seikrit C Tang SCW Boor P Tesar V Floege J et al. IgA nephropathy. *Nat Rev Dis Primers*. (2023) 9:67. 10.1038/s41572-023-00476-9 38036542

[2] Floege J Barratt J Cook HT Noronha IL Reich HN Suzuki Y et al. Executive summary of the KDIGO 2025 clinical practice guideline for the management of immunoglobulin a nephropathy (IgAN) and immunoglobulin a vasculitis (IgAV). *Kidney Int*. (2025) 108:548–54. 10.1016/j.kint.2025.04.003 40975525

[3] Ohyama Y Renfrow MB Novak J Takahashi K. Aberrantly glycosylated IgA1 in IgA nephropathy: what we know and what we don’t know. *J Clin Med*. (2021) 10:3467. 10.3390/jcm10163467 34441764 PMC8396900

[4] Novak L Hall SD Cutter G Gurganus GL Rizk DV Julian BA et al. Kidney injury and colocalization of complement C3, IgA, and IgG in glomerular immune-complex deposits of patients with IgA nephropathy or IgA vasculitis with nephritis. *Kidney Int*. (2025) 108:1158–69. 10.1016/j.kint.2025.07.038 40992572 PMC12626161

[5] Zeng Q Wang WR Li YH Liang Y Wang XH Yan L et al. Diagnostic and prognostic value of galactose-deficient IgA1 in patients with IgA nephropathy: an updated systematic review with meta-analysis. *Front Immunol*. (2023) 14:1209394. 10.3389/fimmu.2023.1209394 37671165 PMC10475574

[6] Vaz de Castro PAS Amaral AA Almeida MG Selvaskandan H Barratt J Simões E et al. Examining the association between serum galactose-deficient IgA1 and primary IgA nephropathy: a systematic review and meta-analysis. *J Nephrol.* (2024) 37:2099–112. 10.1007/s40620-023-01874-8 38427309 PMC11649794

[7] Zhao N Hou P Lv J Moldoveanu Z Li Y Kiryluk K et al. The level of galactose-deficient IgA1 in the sera of patients with IgA nephropathy is associated with disease progression. *Kidney Int*. (2012) 82:790–6. 10.1038/ki.2012.197 22673888 PMC3443545

[8] Shukla M Malhotra KP Chandra A Rao NS Ahmad MK. Correlation of serum galactose-deficient IgA1 and Oxford class in cases of IgA nephropathy. *Arch Pathol Lab Med*. (2024) 148:1244–50. 10.5858/arpa.2023-0190-OA 38289288

[9] Wu J Xie T Zhang Z Liu X Zhou X Zhang Y et al. Absence of glomerular IgA1 deposition despite overexpression of galactose-deficient IgA1 in the B cell c1galt1 knockout mouse. *Kidney Int.* (2026) 10.1016/j.kint.2026.02.034 [Epub ahead of print]. 41905596

[10] Barratt J Lafayette R Kristensen J Stone A Cattran D Floege J et al. Results from part a of the multi-center, double-blind, randomized, placebo-controlled NefIgArd trial, which evaluated targeted-release formulation of budesonide for the treatment of primary immunoglobulin a nephropathy. *Kidney Int*. (2023) 103:391–402. 10.1016/j.kint.2022.09.017 36270561

[11] Wimbury D Muto M Bhachu JS Scionti K Brown J Molyneux K et al. Targeted-release budesonide modifies key pathogenic biomarkers in immunoglobulin a nephropathy: insights from the NEFIGAN trial. *Kidney Int*. (2024) 105:381–8. 10.1016/j.kint.2023.11.003 38008160

[12] Zan J Liu L Li G Zheng H Chen N Wang C et al. Effect of telitacicept on circulating Gd-IgA1 and IgA-containing immune complexes in IgA nephropathy. *Kidney Int Rep*. (2024) 9:1067–71. 10.1016/j.ekir.2024.01.003 38765591 PMC11101733

[13] Lv J Liu L Wang W Wang X Zuraw Q Perkovic V et al. Telitacicept for IgA nephropathy - interim analysis of a phase 3 Trial. *N Engl J Med*. (2026) 394:1916–24. 10.1056/NEJMoa2514415 42127391

[14] Lafayette R Barbour S Israni R Wei X Eren N Floege J et al. A phase 2b, randomized, double-blind, placebo-controlled, clinical trial of atacicept for treatment of IgA nephropathy. *Kidney Int*. (2024) 105:1306–15. 10.1016/j.kint.2024.03.012 38552841

[15] Barratt J Barbour SJ Brenner RM Cooper K Wei X Eren N et al. Long-term results from an open-label extension study of atacicept for the treatment of IgA nephropathy. *J Am Soc Nephrol*. (2025) 36:679–87. 10.1681/ASN.0000000541 39462308 PMC7616790

[16] Kooienga L Lo J Lee EY Kim SG Thomas H Workeneh B et al. Zigakibart demonstrates clinical safety and efficacy in a Phase 1/2 trial of healthy volunteers and patients with IgA nephropathy. *Kidney Int*. (2025) 108:445–54. 10.1016/j.kint.2025.05.006 40482854

[17] Barratt J Lafayette RA Zhang H Tesar V Rovin BH Tumlin JA et al. IgA nephropathy: the lectin pathway and implications for targeted therapy. *Kidney Int*. (2023) 104:254–64. 10.1016/j.kint.2023.04.029 37263354

[18] Trimarchi H Barratt J Cattran DC Cook HT Coppo R Haas M et al. Oxford classification of IgA nephropathy 2016: an update from the IgA nephropathy classification working group. *Kidney Int*. (2017) 91:1014–21. 10.1016/j.kint.2017.02.003 28341274

[19] Zachova K Jemelkova J Kosztyu P Ohyama Y Takahashi K Zadrazil J et al. Galactose-deficient IgA1 B cells in the circulation of iga nephropathy patients carry preferentially lambda light chains and mucosal homing receptors. *J Am Soc Nephrol*. (2022) 33:908–17. 10.1681/ASN.2021081086 35115327 PMC9063893

[20] Liu X Wang L Lin M Jia N Liu G Peng J et al. Multi-omics analyses reveal the pathogenic role of terminal ileum-derived IgA+β7+ cells in IgA nephropathy. *Kidney Int*. (2026) 109:780–95. 10.1016/j.kint.2025.12.026 41571098

[21] Kano T Suzuki H Makita Y Fukao Y Suzuki Y. Nasal-associated lymphoid tissue is the major induction site for nephritogenic IgA in murine IgA nephropathy. *Kidney Int*. (2021) 100:364–76. 10.1016/j.kint.2021.04.026 33961870

[22] Zhu Y He H Sun W Wu J Xiao Y Peng Y et al. IgA nephropathy: gut microbiome regulates the production of hypoglycosilated IgA1 via the TLR4 signaling pathway. *Nephrol Dial Transplant*. (2024) 39:1624–41. 10.1093/ndt/gfae052 38402460 PMC11427068

[23] Makita Y Suzuki H Kano T Takahata A Julian BA Novak J et al. TLR9 activation induces aberrant IgA glycosylation via APRIL- and IL-6-mediated pathways in IgA nephropathy. *Kidney Int*. (2020) 97:340–9. 10.1016/j.kint.2019.08.022 31748116 PMC7372907

[24] Sun Q Zhang Z Zhang H Liu X. Aberrant IgA1 glycosylation in IgA nephropathy: a systematic review. *PLoS One*. (2016) 11:e0166700. 10.1371/journal.pone.0166700 27870872 PMC5117702

[25] Dotz V Visconti A Lomax-Browne HJ Clerc F Hipgrave Ederveen AL Medjeral-Thomas NR et al. O- and N-Glycosylation of serum immunoglobulin a is associated with IgA nephropathy and glomerular function. *J Am Soc Nephrol*. (2021) 32:2455–65. 10.1681/ASN.2020081208 34127537 PMC8722783

[26] Si M Fu J Fang M Lu Y Huang J Li H et al. Lysosome-mediated aggregation of galactose-deficient IgA1 with transferrin receptor 1 links to IgA nephropathy. *Nat Commun*. (2025) 16:5536. 10.1038/s41467-025-60819-w 40593574 PMC12219878

[27] Berthelot L Robert T Vuiblet V Tabary T Braconnier A Dramé M et al. Recurrent IgA nephropathy is predicted by altered glycosylated IgA, autoantibodies and soluble CD89 complexes. *Kidney Int*. (2015) 88:815–22. 10.1038/ki.2015.158 26061544

[28] Chen Q Chen P He R Zan J Shen X Lv J et al. Predictive value of Gd-IgA1, poly-IgA in the treatment of IgA nephropathy with targeted-release formulation budesonide. *Clin Kidney J*. (2025) 18:sfaf203. 10.1093/ckj/sfaf203 40678796 PMC12268327

[29] Fu R Gao J Zhang Y He Z Du X. Poly-IgA immune complex as a monitoring biomarker of response to telitacicept in IgA nephropathy. *Front Immunol*. (2026) 17:1751549. 10.3389/fimmu.2026.1751549 42292347 PMC13260420

[30] Kang Y Xu B Shi S Zhou X Chen P Liu L et al. Mesangial C3 deposition, complement-associated variant, and disease progression in IgA nephropathy. *Clin J Am Soc Nephrol*. (2023) 18:1583–91. 10.2215/CJN.0000000000000290 37651123 PMC10723908

[31] Kesarwani V Bukhari MH Kahlenberg JM Wang S. Urinary complement biomarkers in immune-mediated kidney diseases. *Front Immunol*. (2024) 15:1357869. 10.3389/fimmu.2024.1357869 38895123 PMC11184941

[32] Lafayette RA Rovin BH Reich HN Tumlin JA Floege J Barratt J. Safety, tolerability and efficacy of narsoplimab, a novel MASP-2 inhibitor for the treatment of IgA nephropathy. *Kidney Int Rep*. (2020) 5:2032–41. 10.1016/j.ekir.2020.08.003 33163724 PMC7609886

[33] ClinicalTrials.gov. A Randomized, Double-Blind, Placebo-Controlled, Phase 3 Study of the Safety and Efficacy of OMS721 in Patients With Immunoglobulin a Nephropathy (ARTEMIS-IgAN). Bethesda, MD: U.S. National Library of Medicine (NLM) (2026).

[34] Tang Y Tao X Huang C Teng S Lin X Tian J et al. Urinary IL-18 predicts progression of IgA nephropathy. *BMC Nephrol*. (2026) 27:312. 10.1186/s12882-026-04965-3 41942880 PMC13181966

[35] Cheung CK Barratt J Liew A Zhang H Tesar V Lafayette R. The role of BAFF and APRIL in IgA nephropathy: pathogenic mechanisms and targeted therapies. *Front Nephrol*. (2024) 3:1346769. 10.3389/fneph.2023.1346769 38362118 PMC10867227

[36] Muto M Suzuki H Suzuki Y. New insights and future perspectives of APRIL in IgA Nephropathy. *Int J Mol Sci*. (2024) 25:10340. 10.3390/ijms251910340 39408691 PMC11476402

[37] Lafayette R Barbour SJ Brenner RM Campbell KN Doan T Eren N et al. A Phase 3 Trial of atacicept in patients with IgA nephropathy. *N Engl J Med*. (2026) 394:647–57. 10.1056/NEJMoa2510198 41196369

[38] Cincinnati Children’s Nephrology Clinical Laboratory. *Gd-IgA1 Biomarker Screening Now Available. Cincinnati Children’s Hospital Medical Center.* (2025). Available online at: https://www.cincinnatichildrens.org/-/media/Cincinnati-Childrens/Home/clinical-labs/nephrology/Gd-IgA1-Biomarker-Screening.pdf (accessed August 20, 2026).

[39] Mayo Clinic Laboratories. *Galactose Deficient IgA1 (KM55) Immunostain, Technical Component Only.* (2026). Available online at: https://www.mayocliniclabs.com/test-catalog/overview/621066 (accessed August 20, 2026).

[40] Suzuki K Honda K Tanabe K Toma H Nihei H Yamaguchi Y. Incidence of latent mesangial IgA deposition in renal allograft donors in Japan. *Kidney Int*. (2003) 63:2286–94. 10.1046/j.1523-1755.63.6s.2.x 12753320

[41] Wang Z Zhang X Han W Yu G Ying Z Xu X et al. Immune characteristics of renal allograft donors with mesangial IgA deposition. *Int Immunopharmacol*. (2021) 91:107282. 10.1016/j.intimp.2020.107282 33370682

[42] Barbour SJ Coppo R Zhang H Liu ZH Suzuki Y Matsuzaki K et al. Evaluating a new international risk-prediction tool in IgA nephropathy. *JAMA Intern Med*. (2019) 179:942–52. 10.1001/jamainternmed.2019.0600 30980653 PMC6583088

